# Predictors of cinacalcet discontinuation and reinitiation in hemodialysis patients: results from 7 European countries

**DOI:** 10.1186/s12882-019-1355-5

**Published:** 2019-05-14

**Authors:** Douglas S. Fuller, David Hallett, Paul J. Dluzniewski, Bruno Fouqueray, Michel Jadoul, Hal Morgenstern, Friedrich K. Port, Francesca Tentori, Ronald L. Pisoni

**Affiliations:** 1 Arbor Research Collaborative for Health, Ann Arbor, MI USA; 2 Amgen, Inc., Thousand Oaks, CA USA; 3 Amgen (Europe) GmbH, Rotkreuz, Switzerland; 4 Cliniques Universitaires St-Luc, Université catholique de Louvain, Bruxelles, Belgium; 5 Departments of Epidemiology and Environmental Health Sciences, School of Public Health, and Department of Urology, Medical School, University of Michigan, Ann Arbor, MI USA; 6 DaVita, Inc., Minneapolis, MN USA; 7 Vanderbilt University School of Medicine, Nashville, TN USA

**Keywords:** Cinacalcet, Hemodialysis, Parathyroid hormone

## Abstract

**Background:**

The putative benefits of cinacalcet therapy for management of secondary hyperparathyroidism (SHPT) are thought to be most manifested when patients are taking it consistently and as prescribed. Real-world descriptions of cinacalcet prescription discontinuation and reinitiation in European hemodialysis patients are lacking. To address this knowledge gap, we used Dialysis Outcomes and Practice Patterns Study (DOPPS) data, based on dialysis facility medical records, from seven European countries to estimate rates and predictors of cinacalcet prescription discontinuation and reinitiation in hemodialysis patients and to describe the trajectories of CKD-MBD laboratory values after discontinuation.

**Methods:**

Cox regression analyses were used to predict (1) cinacalcet discontinuation among 613 patients with ≥3 consecutive months without cinacalcet prescription immediately prior to a new cinacalcet prescription and (2) cinacalcet reinitiation among 415 patients with a newly discontinued cinacalcet prescription immediately after ≥3 consecutive months of prescribed use.

**Results:**

Cinacalcet was discontinued in 21 and 35% of new users after 6 and 12 months, respectively. Cinacalcet was reinitiated in 38 and 49% of newly-discontinued users after 6 and 12 months, respectively. Predictors of discontinuation included lower parathyroid hormone (PTH) in the previous month (< 150 pg/ml vs. 150–299, HR = 2.57 [95% CI: 1.52–4.33]) and lower serum calcium in the previous month (< 8.4 mg/dl vs. 8.4–10.19, HR = 1.67 [95% CI: 1.08–2.59]). Predictors of reinitiation included higher PTH in the previous month (300–599 pg/ml vs. 150–299, HR = 1.88 [95% CI = 1.19–2.97]; 600+ pg/ml, HR = 3.02 [95% CI = 1.92–4.76]). After cinacalcet discontinuation, mean serum PTH increased from 408 to 510 pg/ml, mean serum calcium briefly rose from 9.12 to 9.22 mg/dl before declining to 9.06 mg/dl, and mean serum phosphorus showed little change.

**Conclusions:**

Nephrologist discontinuation of cinacalcet therapy is common in European countries. Additional research is needed to identify optimal cinacalcet treatment strategies for SHPT management, including comparisons of intermittent cinacalcet therapy versus sustained treatment with reduced dose or frequency.

**Electronic supplementary material:**

The online version of this article (10.1186/s12882-019-1355-5) contains supplementary material, which is available to authorized users.

## Background

Secondary hyperparathyroidism (SHPT) is a common complication of chronic kidney disease (CKD) and end-stage renal disease (ESRD) that can lead to elevated parathyroid hormone (PTH), serum calcium, and serum phosphorus levels [1–3]. Estimates of SHPT prevalence vary substantially, with PTH levels > 300 pg/ml in 30–50% of hemodialysis patients in most countries [4]. SHPT is associated with increased risks of fractures, cardiovascular disease, and mortality in dialysis patients [5–7] and is typically managed using a variety of treatments, including changes to dietary intake or dialysis prescription, vitamin D compounds, calcimimetics, and phosphate binders [8, 9].

Cinacalcet (Amgen Inc., Thousand Oaks, CA United States [10]) is an oral calcimimetic agent used in approximately 15–20% of the European hemodialysis population [7, 11]. It directly reduces PTH levels with an additional resultant effect of decreasing serum calcium and phosphorus levels [12, 13]. As with most chronically administered medications, the putative benefits of cinacalcet therapy are thought to manifest when patients are taking it consistently as prescribed, and consequently, the benefits of cinacalcet may not be fully realized when not remaining on therapy [14].

Medication non-adherence – failure to follow timing, dosage, or frequency recommendations –precludes patients from attaining the greatest benefits of their prescribed therapy, and has been associated with increased mortality, hospitalizations, and health care costs [15–17]. A very broad range in medication non-adherence has been estimated in hemodialysis patients [18], with estimates ranging from 3 to 80% across 19 studies [19], and 13 to 99% across 44 studies [20]. Although medication non-adherence can be difficult to accurately measure, it is meaningful to understand variations across nephrologists in rates of starting and stopping cinacalcet prescriptions, and related factors. Despite evidence of the benefits of managing SHPT-related biochemistries with cinacalcet, post-marketing studies in real-world settings - largely in the US - indicate that treatment discontinuation is common [21–24], and is associated with demographic, clinical, and financial factors.

Compared to those in the US, most studies of cinacalcet discontinuation in the European dialysis population have been more limited in size and scope. In a retrospective cohort study of 5193 incident European hemodialysis patients receiving treatment from a single large-chain provider, the authors reported that 23% of cinacalcet users discontinued treatment (defined as ≥45 consecutive prescription-free days) during a maximum of 12 months of follow-up [25]. Additional work is needed to obtain a broader understanding of real-world cinacalcet discontinuation in Europe, associated factors, and fluctuations in PTH, calcium, and phosphorus levels following discontinuation – particularly in view of the differing health care delivery structures for hemodialysis patients across various European countries.

To help inform this knowledge gap and inform key aspects of real world cinacalcet prescription in hemodialysis patients, we have examined the rates of European nephrologists discontinuing and re-initiating cinacalcet prescriptions for their hemodialysis patients, along with assessing numerous factors to understand key predictors of cinacalcet discontinuation and reinitiation prescriptions by European nephrologists. Our analyses are based upon data abstracted from dialysis facility medical records in national samples of hemodialysis facilities and patients from seven European countries participating in the Dialysis Outcomes and Practice Patterns Study (DOPPS). Our study goals are to: (1) describe rates of cinacalcet prescription discontinuation and reinitiation based upon a patient’s medical record, (2) identify factors associated with cinacalcet prescription discontinuation and reinitiation, and (3) describe the trajectories of PTH, calcium, and phosphorus laboratory values following cinacalcet prescription discontinuation.

## Methods

### Data source

The study population for our analysis was drawn from all adult patients receiving center-based hemodialysis at a DOPPS participating facility during phases 4 (2009–2011) and 5 (2012–2015) from Belgium (phase 4 only), France, Germany, Italy, Spain, Sweden (phase 4 only), and the United Kingdom (UK). DOPPS 5 follow-up time in France and Spain was reduced due to a mid-phase change in study protocol. DOPPS was approved by a central institutional review board (IRB) in the US, with additional IRB study approval and patient consent obtained to meet national and local ethics committee regulations at each study site. Patients were required to survive at least 4 months after enrollment in their respective DOPPS phase; this minimum survival requirement ensured sufficient determination of cinacalcet non-exposure enabling the retrospective identification of new (not necessarily first) users, and allowed for the measurement of baseline covariates.

Detailed clinical and laboratory data were collected monthly from dialysis facility medical records. In phase 4, prescription status for cinacalcet and other renal medications was indicated on the dialysis facility’s patient medication list as of the last day of each study month; in phase 5, prescription status was indicated based upon any active prescription for cinacalcet on the medication list during each study month. Single months during follow-up with missing cinacalcet prescription data used the prior month value (i.e., last -observation -carry-forward; < 5% of months). Follow-up periods containing two consecutive months with missing cinacalcet prescription data were censored as of the first missing month.

### Cohort identification

**Fig. 1:**
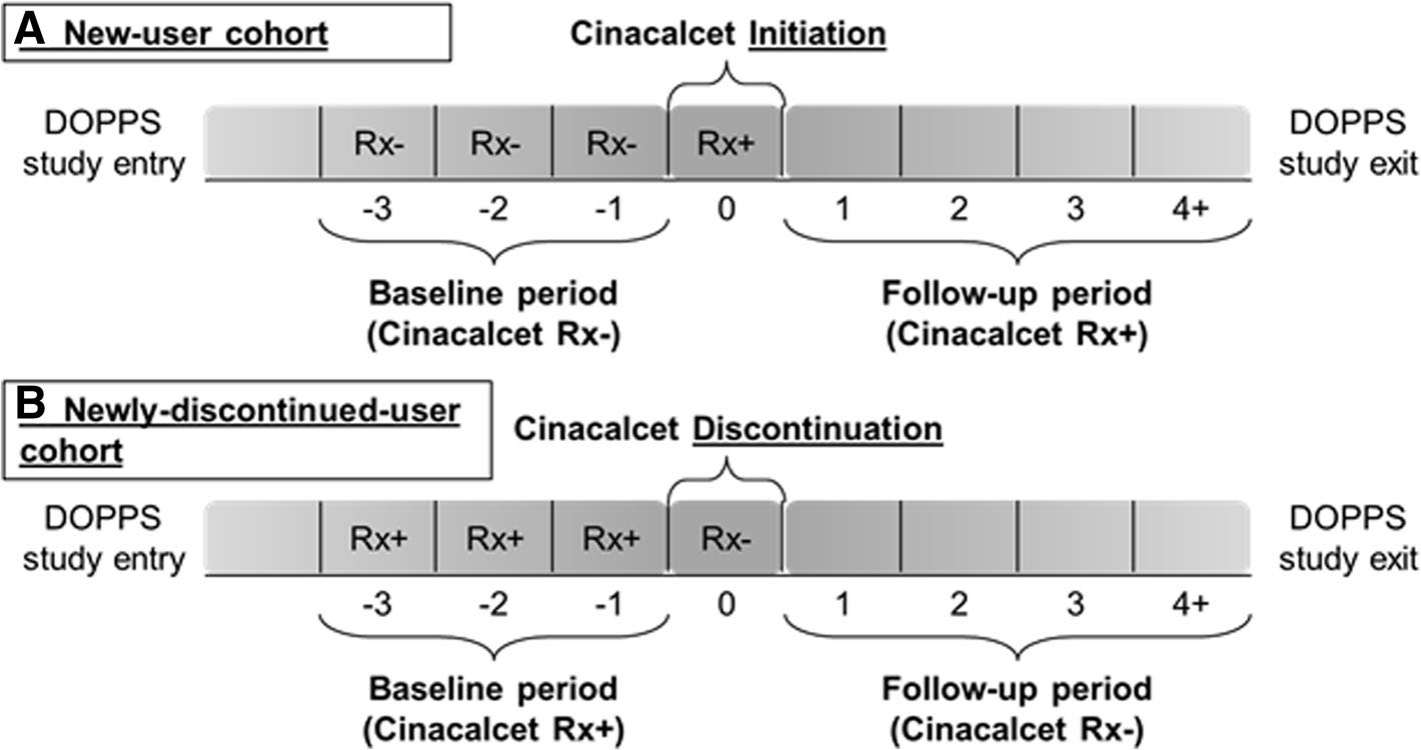
Cohort identification diagram. Panel **a** denotes the treatment pattern required for entry into the "new-user" analysis cohort. Panel **b** denotes the treatment pattern required for entry into the "newly-discontinued-user" analysis cohort

Patients eligible for the analysis of cinacalcet discontinuation (“new-user cohort”) were defined as having a period of at least 3 consecutive months without cinacalcet prescription (baseline period) immediately prior to a month containing a new cinacalcet prescription (this month is referred to as the ‘index’ month; Fig. 1a). Follow-up time began with the first monthly record after the index month and continued until the cinacalcet prescription was discontinued (i.e., removed from the medication list). Follow-up was censored if the patient received a parathyroidectomy or was lost to follow-up (i.e. due to death or departure from the study facility), or DOPPS follow-up was terminated for administrative reasons. Patients with parathyroidectomy prior to start of follow-up were excluded.

Patients eligible for the analysis of reinitiation after discontinuation and post-discontinuation trajectories of CKD-mineral and bone disorder (MBD) lab markers (“newly-discontinued-user cohort”) were defined as having a period of at least 3 consecutive months with a cinacalcet prescription immediately prior to a month in which the prescription was discontinued (i.e., ‘index’ month; Fig. 1b). Follow-up time began with the first monthly record after the index month and continued until a new cinacalcet prescription was started (i.e., added to the medication list). Follow-up was censored if the patient received a parathyroidectomy or was lost to follow-up (i.e. due to death or departure from study facility), or DOPPS follow-up was terminated. Patients with parathyroidectomy prior to start of follow-up were excluded. We additionally conducted two sensitivity analyses to explore alternative definitions for the newly-discontinued-user cohort: (1) reducing the baseline period to 1 month of cinacalcet prescription, instead of 3 months, prior to discontinuation, and (2) restricting to patients in the new-user cohort (defined above) who experienced a discontinuation. No substantial differences in reinitiation rates were observed using these alternative definitions, so we reported only the primary results.

### Statistical analyses

We estimated crude (unadjusted) discontinuation and reinitiation rates by dividing the number of first discontinuation or reinitiation events by the total number of follow-up months at risk of each outcome. Rate estimates are expressed per month, and are provided overall and by country. We additionally report the proportion of patients discontinuing or resuming cinacalcet treatment at each follow-up month using Kaplan-Meier methods. Comparisons of these curves were carried out using log-rank tests.

Demographic, clinical history, and treatment variables were ascertained at a single time during the baseline period (defined above for each cohort) or updated during follow-up using a 1-month lag (“time-dependent”) to ensure temporal precedence of predictor values with respect to outcomes. For time-dependent covariates with missing data, we imputed intermittent missing values encountered during follow-up periods for up to 2 consecutive months using the last-observation-carry-forward method. No values were imputed for months that occurred after a subject ceased study observation. This method is consistent with clinical treatment decision-making in regard to infrequently measured variables.

We examined crude and adjusted associations of discontinuation and reinitiation with demographic and clinical factors using Cox proportional hazards regression models. Non-proportionality was assessed using standard graphical and statistical methods. Baseline covariates adjusted for in models were age, years with ESRD (vintage), sex, body mass index (BMI) and 13 summary comorbid conditions (coronary artery disease, congestive heart failure, other cardiovascular disease, cancer [other than skin], diabetes, hypertension, recent GI bleeding, psychiatric disorder [depression, bipolar disorder, schizophrenia, and alcohol or substance abuse], peripheral vascular disease, lung disease, neurologic disorder, recurrent cellulitis, cerebrovascular disease). CKD-MBD laboratory values (serum levels of calcium, phosphorus, and PTH), clinical status measures (hospitalization, serum albumin level) and concurrent prescriptions of related CKD-MBD treatments (vitamin D, phosphate binders) were measured at baseline and during follow-up as time-dependent variables. Continuous variables were categorized using clinically meaningful cut-points.

Among patients in the newly-discontinued-user cohort, we estimated the trajectories of CKD-MBD laboratory values (calcium, phosphorus, PTH) for 12 months in two ways: (1) censoring follow-up at reinitiation of cinacalcet treatment (prescription) or loss to follow-up for any reason (*n* = 367); and (2) restricting to patients with 12 months of cinacalcet-free follow-up (*n* = 81). We used LOESS (LOcal regrESSion) curves to depict the smoothed trend, and 95% confidence limits for the means are reported.

## Results

### Cinacalcet discontinuation in the new-user cohort

**Fig. 2:**
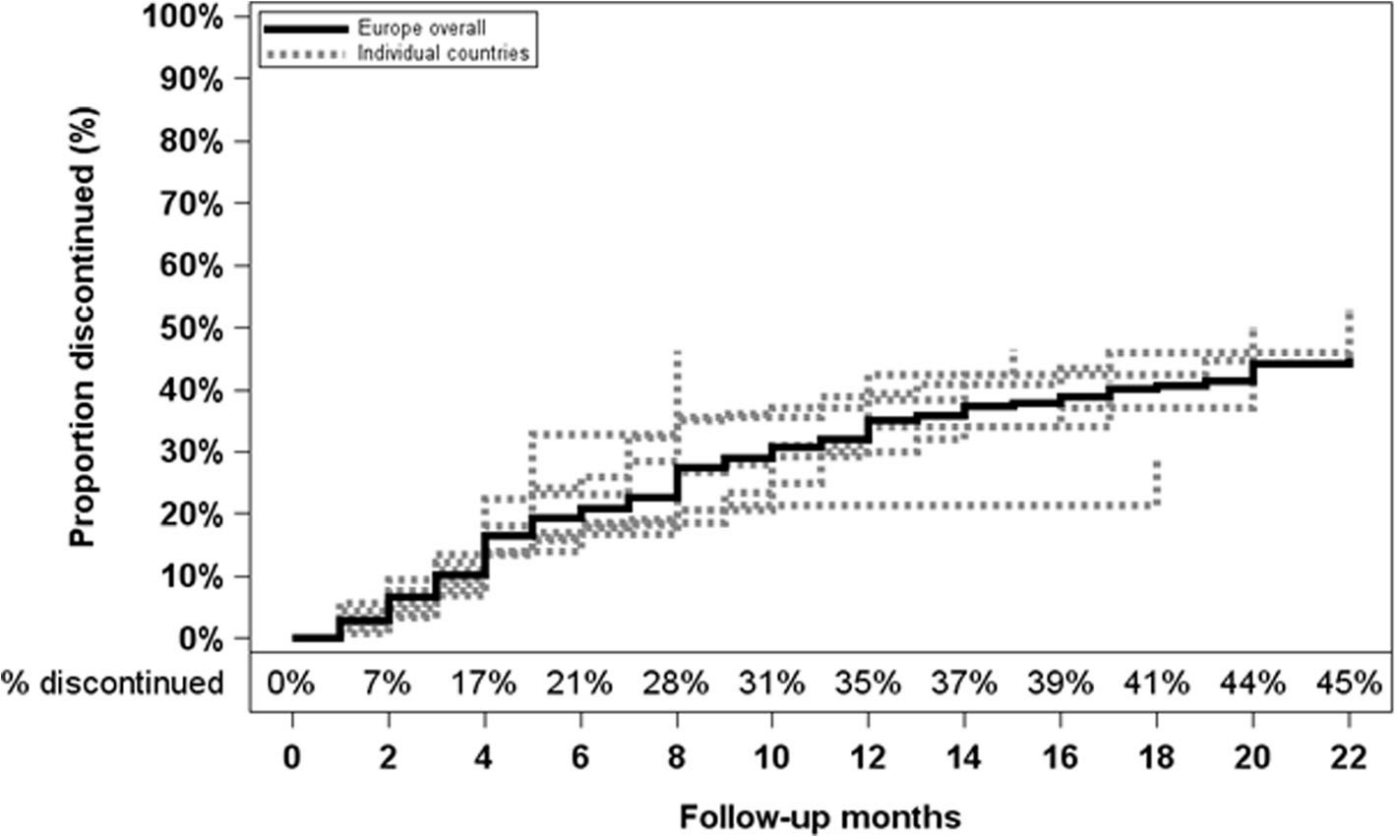
Proportion of patients *discontinuing* cinacalcet during follow-up (new-user cohort; *n* = 613). The solid line represents Europe overall, and the dashed lines denote individual countries

Overall, new cinacalcet prescriptions were initiated in 613 hemodialysis patients during the study, and these patients contributed 5454 months of follow-up to the discontinuation analysis. Therapy was discontinued in 172 patients (Table 1, left panel). The overall discontinuation rate was 0.032/month and ranged two-fold by country, from 0.021/month in Sweden to 0.045/month in Belgium. The overall proportion of patients discontinuing cinacalcet was 21% after 6 months and 35% after 12 months; variability among countries at 12 months ranged from 21% in Sweden to 46% in Belgium (Fig. 2). Patients who discontinued cinacalcet had a higher prevalence of heart- and vascular-related comorbid conditions, but lower median PTH levels at initiation [430 pg/mL (IQR: 317,749)] compared to that for patients who did not discontinue cinacalcet [507 pg/mL (IQR: 340,780)] (Table 2).

**Table 1:** Crude rates of cinacalcet discontinuation and reinitiation, overall and by country

	Discontinuation in new-user cohort				Reinitiation in newly-discontinued-user cohort			
	N	Months at risk	Discontinuations	Crude rate^a^	N	Months at risk	Reinitiations	Crude rate^a^
Europe	613	5454	172	0.032	415	2964	171	0.058
Belgium	18	112	5	0.045	14	104	5	0.048
France	60	462	13	0.028	34	207	11	0.053
Germany	108	1128	30	0.027	74	617	30	0.049
Italy	149	1465	53	0.036	140	1129	67	0.059
Spain	147	1192	41	0.034	85	472	27	0.057
Sweden	68	580	12	0.021	30	196	9	0.046
UK	63	515	18	0.035	38	239	22	0.092

^a^Each crude rate was computed as the number of outcome events observed during follow-up (discontinuations or reinitiations), divided by the number of person-months at risk. Thus, each rate is expressed per month

**Table 2:** Selected characteristics of patients at start of follow-up, by analysis cohort

	New-user cohort		Newly-discontinued-user cohort	
	Discontinued cinacalcet	Did not discontinue	Reinitiated cinacalcet	Did not reinitiate
N	172	441	171	244
Age (yrs)	62.8 (16.9)	61.9 (15.6)	63.6 (14.8)	63.5 (15.8)
Time on dialysis (yrs) [median/IQR]	3.9 [1.8, 7.1]	3.7 [1.7, 7.0]	5.1 [2.7, 9.6]	4.5 [2.4, 8.5]
Male (%)	60.5	62.1	52.0	58.9
Coronary artery disease (%)	31.6	26.1	28.2	32.9
Cancer (%)	16.4	11.7	14.8	12.6
Other cardiovascular disease (%)	24.0	27.1	25.9	28.9
Cerebrovascular disease (%)	16.5	11.2	13.6	14.2
Congestive heart failure (%)	15.8	12.2	12.9	15.5
Diabetes (%)	28.7	29.6	27.8	31.1
GI Bleeding (%)	2.9	3.4	4.1	5.0
Hypertension (%)	86.5	88.5	87.6	86.3
Lung disease (%)	16.5	11.9	9.5	15.5
Psychiatric disorder (%)	21.6	11.5	17.1	21.8
Peripheral vascular disease (%)	31.6	24.8	27.6	30.1
Hospitalized in baseline period (%)	18.0	16.1	15.8	18.7
BMI (kg/m^2^)	25.9 (5.3)	26.5 (5.2)	26.2 (5.3)	26.2 (5.4)
Serum phosphorus (mg/dl)	5.3 (1.7)	5.4 (1.5)	5.1 (1.7)	5.1 (1.6)
Serum PTH (pg/ml) [median/IQR]	430 [317, 749]	507 [340, 780]	310 [145, 575]	237 [117, 518]
Serum total calcium (mg/dl)	9.1 (0.9)	9.3 (0.8)	9.0 (0.9)	8.9 (0.8)
Serum albumin (g/dl)	3.8 (0.4)	3.8 (0.5)	3.8 (0.5)	3.7 (0.5)
Phosphate binder use (%)				
No phosphate binder use	17.5	15.8	15.5	18.0
Ca-containing only	7.0	12.3	13.1	11.7
Non-Ca-containing only	57.9	53.3	54.2	49.4
Both	17.5	18.6	17.3	20.9
Vitamin D (active/analog) use (%)				
No vitamin D use	42.9	37.5	34.5	42.3
IV vitamin D only	29.4	30.1	31.6	27.2
Oral vitamin D only	25.9	32.4	32.1	29.3
Both	1.8	0.0	1.8	1.3

New-user cohort patients required at least 3 consecutive months without cinacalcet prescription prior to starting cinacalcet. Newly-discontinued-user cohort patients required at least 3 consecutive months with cinacalcet prescription prior to discontinuing cinacalcet. Values represent mean (standard error) unless noted otherwise. Comorbidities were measured at DOPPS study entry; all other values were measured at the end of the baseline period prior to the start of follow-up for each cohort. Psychiatric disorder includes depression, bipolar disorder, schizophrenia, and alcohol or substance abuse

Adjusted associations between cinacalcet discontinuation and most baseline case-mix variables were highly variable. A higher adjusted hazard ratio for discontinuation was observed for patients with psychiatric disorder (HR = 1.75 [95% CI = 1.15–2.66]) (Table 3). In time-varying Cox regression analyses, higher adjusted hazard ratios for discontinuation were observed for lower PTH in the previous month (< 150 pg/ml vs. 150–299, HR = 2.57 [95% CI = 1.52–4.33]) and lower serum calcium levels in the previous month (7.50–8.39 mg/dl vs. 8.40–10.19, HR = 1.65 [95% CI = 1.04–2.60]; < 7.50, HR = 1.83 [95% CI = 0.83–4.04]). Patients with low serum phosphorus in the previous month (< 3.50 vs 3.50–5.49, HR = 1.58 [95% CI = 1.09–2.30]) or who were hospitalized in the prior 3 months (HR = 1.40; 95% CI = 1.01–1.92) were also more likely to have cinacalcet prescription discontinued.

**Table 3:** Estimated effects (crude and adjusted hazard ratios [HR] and 95% confidence intervals [CI]) of patient predictors on cinacalcet discontinuation, by type of predictor (baseline or time-dependent), in the new-user cohort (*n* = 613)

	Baseline				Time-dependent			
	Crude		Adjusted^a^		Crude		Adjusted ^a^	
Variable	HR	95% CI	HR	95% CI	HR	95% CI	HR	95% CI
Age (yrs)								
< 45	1.20	(0.74, 1.92)	1.36	(0.81, 2.30)				
45–54	0.78	(0.43, 1.39)	0.83	(0.46, 1.49)				
55–64	1	(ref)	1	(ref)				
65–74	0.97	(0.65, 1.47)	0.85	(0.52, 1.40)				
75+	1.48	(0.96, 2.26)	1.36	(0.83, 2.22)				
Vintage (yrs)								
< 1.0	0.78	(0.43, 1.42)	0.79	(0.44, 1.42)				
1.0–2.9	1	(ref)	1	(ref)				
3.0–4.9	1.16	(0.78, 1.71)	1.09	(0.74, 1.63)				
5.0+	0.92	(0.64, 1.33)	0.87	(0.59, 1.28)				
BMI (kg/m^2^)								
< 18.5	1.49	(0.75, 2.96)	1.31	(0.58, 2.97)				
18.5–24.9	1.44	(1.01, 2.07)	1.56	(1.05, 2.30)				
25.0–29.9	1	(ref)	1	(ref)				
30.0+	1.17	(0.74, 1.85)	1.30	(0.80, 2.12)				
Male sex	0.98	(0.73, 1.32)	0.94	(0.67, 1.30)				
Coronary artery disease	1.48	(1.08, 2.03)	1.26	(0.90, 1.74)				
Cancer	1.40	(0.95, 2.04)	1.31	(0.91, 1.89)				
Other cardiovascular disease	1.20	(0.80, 1.79)	1.06	(0.67, 1.68)				
Cerebrovascular disease	1.53	(1.03, 2.27)	1.39	(0.93, 2.06)				
Congestive heart failure	1.51	(1.02, 2.25)	1.21	(0.79, 1.85)				
Diabetes	0.99	(0.71, 1.38)	0.87	(0.57, 1.32)				
GI bleeding	0.94	(0.47, 1.86)	0.76	(0.33, 1.73)				
Hypertension	0.88	(0.59, 1.30)	0.77	(0.48, 1.23)				
Lung disease	1.30	(0.83, 2.03)	1.15	(0.73, 1.80)				
Psychiatric disorder	1.91	(1.29, 2.81)	1.75	(1.15, 2.66)				
Peripheral vascular disease	1.40	(1.00, 1.95)	1.24	(0.88, 1.74)				
Hospitalization in prior 3 months	1.26	(0.85, 1.87)	1.21	(0.79, 1.84)	1.58	(1.13, 2.20)	1.40	(1.01, 1.92)
Serum phosphorus (mg/dl)								
< 3.50	1.31	(0.78, 2.20)	1.39	(0.78, 2.49)	1.61	(1.11, 2.34)	1.58	(1.09, 2.30)
3.50–5.49	1	(ref)	1	(ref)	1	(ref)	1	(ref)
5.50–5.99	1.50	(1.02, 2.19)	1.43	(0.97, 2.11)	1.43	(0.87, 2.35)	1.36	(0.83, 2.21)
6.00+	0.97	(0.70, 1.35)	1.03	(0.73, 1.47)	1.05	(0.70, 1.58)	1.04	(0.69, 1.57)
Serum PTH (pg/ml)								
< 150	2.90	(1.52, 5.12)	2.26	(1.24, 4.12)	2.24	(1.39, 3.59)	2.57	(1.52, 4.33)
150–299	1	(ref)	1	(ref)	1	(ref)	1	(ref)
300–599	1.51	(0.90, 2.55)	1.47	(0.86, 2.52)	0.77	(0.48, 1.25)	0.84	(0.52, 1.38)
600+	1.60	(0.92, 2.81)	1.50	(0.85, 2.64)	0.93	(0.58, 1.50)	0.96	(0.58, 1.57)
Serum calcium (mg/dl)								
< 7.50	1.51	(0.67, 3.39)	1.43	(0.56, 3.68)	1.97	(0.89, 4.38)	1.83	(0.83, 4.04)
7.50–8.39	1.54	(1.03, 2.31)	1.56	(0.99, 2.45)	1.68	(1.11, 2.54)	1.65	(1.04, 2.60)
8.40–10.19	1	(ref)	1	(ref)	1	(ref)	1	(ref)
10.20+	0.91	(0.55, 1.50)	0.90	(0.52, 1.55)	1.06	(0.57, 1.99)	1.03	(0.52, 2.06)
Serum albumin (g/dl)								
< 3.20	0.91	(0.49, 1.67)	0.82	(0.46, 1.46)	1.26	(0.69, 2.29)	1.22	(0.69, 2.15)
3.20–3.49	1.17	(0.74, 1.85)	1.10	(0.67, 1.80)	0.98	(0.57, 1.68)	0.96	(0.55, 1.67)
3.50–3.79	1.14	(0.81, 1.61)	1.16	(0.81, 1.66)	1.08	(0.75, 1.54)	1.19	(0.80, 1.76)
3.80+	1	(ref)	1	(ref)	1	(ref)	1	(ref)
Phosphate binder use								
None	1	(ref)	1	(ref)	1	(ref)	1	(ref)
Ca-containing only	0.60	(0.28, 1.31)	0.51	(0.25, 1.08)	0.75	(0.38, 1.51)	0.54	(0.27, 1.08)
Non-Ca-containing only	0.97	(0.62, 1.54)	0.91	(0.58, 1.44)	1.00	(0.65, 1.56)	0.94	(0.60, 1.49)
Both	0.97	(0.56, 1.69)	0.79	(0.45, 1.40)	0.79	(0.44, 1.40)	0.64	(0.36, 1.13)
Vitamin D use								
None	1	(ref)	1	(ref)	1	(ref)	1	(ref)
IV vitamin D only	0.95	(0.66, 1.38)	1.00	(0.66, 1.51)	1.30	(0.91, 1.85)	1.36	(0.91, 2.03)
Oral vitamin D only	0.76	(0.51, 1.15)	0.83	(0.56, 1.22)	1.17	(0.76, 1.79)	1.12	(0.70, 1.79)

^a^Adjusted for baseline covariates: age, vintage, 13 summary comorbid conditions, body mass index, and male sex. Separate models estimated for each variable shown. Time-dependent effects estimated only for variables shown. Psychiatric disorder includes depression, bipolar disorder, schizophrenia, and alcohol or substance abuse

### Cinacalcet reinitiation in the newly-discontinued-user cohort

**Fig. 3:**
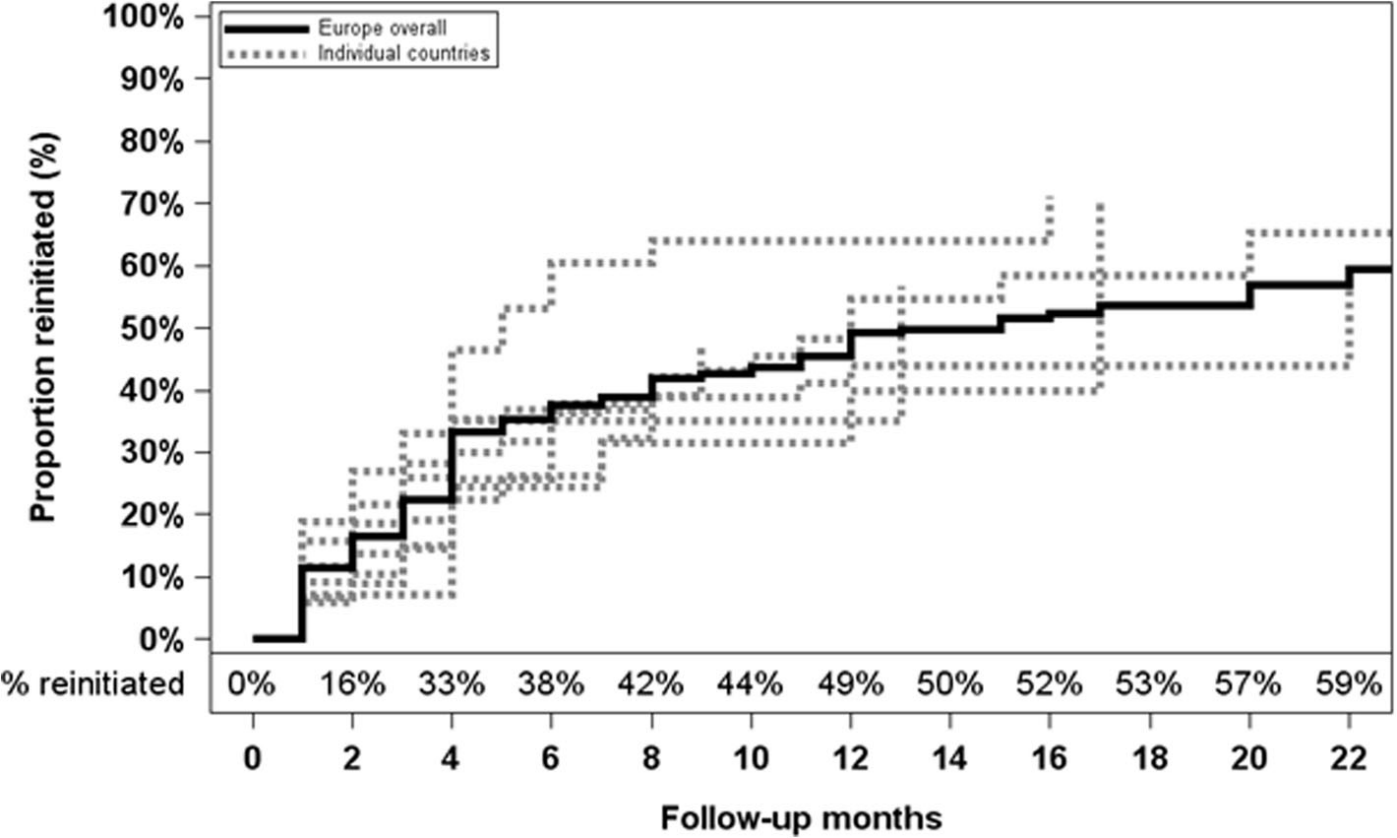
Proportion of patients *reinitiating* cinacalcet during follow-up (newly-discontinued-user cohort; *n* = 415). The solid line represents Europe overall, and the dashed lines denote individual countries

We identified 415 patients in our newly-discontinued-user cohort. During 2964 months of follow-up, cinacalcet was reinitiated in 171 patients (Table 1, right panel). The overall reinitiation rate was 0.058/month and ranged two-fold across countries, from 0.046/month in Sweden to 0.092/month in the UK. The overall proportion of patients reinitiating cinacalcet was 38% after 6 months and 49% after 12 months; variability across countries at 12 months ranged from 35% in Belgium to 64% in the UK (Fig. 3). Patients who reinitiated cinacalcet treatment after a discontinuation had longer median dialysis vintage, were more likely to be female, and lower % with lung disease, but higher median PTH levels at start of discontinuation [310 (IQR: 145, 575) vs 237 (IQR: 117, 518) pg/ml].

Baseline variables were not strongly associated with cinacalcet reinitiation, except for high calcium levels (10.20+ mg/dl vs. 8.4–10.19, HR = 2.04 [95% CI = 1.28–3.25]) (Table 4). Lower reinitiation rates were associated with very low values in the prior month of serum phosphorus (< 3.50 mg/dl vs. 3.50–5.49, HR = 0.40 [95% CI = 0.24–0.66]) and albumin (< 3.20 g/dl vs. 3.80+, HR = 0.49 [95% CI = 0.26–0.92]). Higher reinitiation rates were most strongly associated with higher PTH in the previous month (300–599 pg/ml vs. 150–299, HR = 1.88 [95% CI = 1.19–2.97]; 600+ pg/ml, HR = 3.02 [95% CI = 1.92–4.76]).

**Table 4:** Estimated effects (crude and adjusted hazard ratios [HR] and 95% confidence intervals [CI]) of patient predictors on cinacalcet reinitiation, by type of predictor (baseline or time-dependent), in the newly-discontinued-user cohort (*n* = 415)

	Baseline				Time-dependent			
	Crude		Adjusted^a^		Crude		Adjusted ^a^	
Variable	HR	95% CI	HR	95% CI	HR	95% CI	HR	95% CI
Age (yrs)								
< 45	0.91	(0.53, 1.54)	0.87	(0.49, 1.54)				
45–54	0.90	(0.57, 1.42)	1.01	(0.60, 1.71)				
55–64	1	(ref)	1	(ref)				
65–74	0.98	(0.64, 1.51)	0.96	(0.58, 1.61)				
75+	1.05	(0.71, 1.56)	0.93	(0.59, 1.45)				
Vintage (yrs)								
< 1.0	1.35	(0.74, 2.46)	1.70	(0.86, 3.37)				
1.0–2.9	1	(ref)	1	(ref)				
3.0–4.9	1.17	(0.76, 1.80)	1.26	(0.77, 2.07)				
5.0+	1.23	(0.79, 1.92)	1.25	(0.76, 2.05)				
BMI (kg/m^2^)								
< 18.5	1.20	(0.54, 2.65)	1.21	(0.54, 2.71)				
18.5–24.9	1.21	(0.82, 1.78)	1.19	(0.79, 1.79)				
25.0–29.9	1	(ref)	1	(ref)				
30.0+	1.00	(0.69, 1.46)	1.08	(0.71, 1.63)				
Male sex	0.90	(0.68, 1.19)	0.84	(0.58, 1.22)				
Coronary artery disease	0.95	(0.67, 1.34)	0.90	(0.59, 1.38)				
Cancer	1.32	(0.86, 2.04)	1.28	(0.78, 2.09)				
Other cardiovascular disease	0.96	(0.72, 1.30)	0.98	(0.70, 1.35)				
Cerebrovascular disease	1.23	(0.78, 1.94)	1.18	(0.69, 2.00)				
Congestive heart failure	0.96	(0.61, 1.51)	0.99	(0.58, 1.69)				
Diabetes	0.92	(0.65, 1.29)	0.99	(0.69, 1.41)				
GI bleeding	0.89	(0.45, 1.78)	0.94	(0.41, 2.16)				
Hypertension	1.06	(0.72, 1.56)	1.23	(0.79, 1.90)				
Lung disease	0.77	(0.51, 1.18)	0.73	(0.45, 1.19)				
Psychiatric disorder	0.92	(0.62, 1.35)	0.94	(0.63, 1.41)				
Peripheral vascular disease	1.11	(0.80, 1.54)	1.21	(0.83, 1.75)				
Hospitalization in prior 3 months	0.83	(0.54, 1.28)	0.76	(0.45, 1.30)	0.77	(0.52, 1.14)	0.82	(0.54, 1.24)
Serum phosphorus (mg/dl)								
< 3.50	0.86	(0.58, 1.28)	0.84	(0.54, 1.31)	0.40	(0.24, 0.67)	0.40	(0.24, 0.66)
3.50–5.49	1	(ref)	1	(ref)	1	(ref)	1	(ref)
5.50–5.99	0.75	(0.41, 1.36)	0.68	(0.40, 1.18)	1.42	(0.91, 2.24)	1.27	(0.85, 1.89)
6.00+	1.06	(0.76, 1.49)	1.03	(0.72, 1.49)	1.01	(0.69, 1.49)	1.05	(0.70, 1.56)
Serum PTH (pg/ml)								
< 150	0.90	(0.60, 1.33)	0.87	(0.54, 1.41)	1.35	(0.80, 2.27)	1.39	(0.84, 2.31)
150–299	1	(ref)	1	(ref)	1	(ref)	1	(ref)
300–599	1.26	(0.81, 1.96)	1.39	(0.84, 2.31)	1.77	(1.15, 2.71)	1.88	(1.19, 2.97)
600+	1.19	(0.75, 1.87)	1.23	(0.72, 2.09)	2.99	(1.91, 4.69)	3.02	(1.92, 4.76)
Serum calcium (mg/dl)								
< 7.50	0.60	(0.17, 2.11)	0.66	(0.18, 2.38)	0.51	(0.17, 1.51)	0.49	(0.15, 1.62)
7.50–8.39	1.13	(0.78, 1.63)	1.15	(0.78, 1.70)	1.05	(0.70, 1.56)	1.03	(0.67, 1.58)
8.40–10.19	1	(ref)	1	(ref)	1	(ref)	1	(ref)
10.20+	2.06	(1.32, 3.20)	2.04	(1.28, 3.25)	1.26	(0.84, 1.88)	1.24	(0.80, 1.93)
Serum albumin (g/dl)								
< 3.20	0.64	(0.40, 1.02)	0.61	(0.36, 1.02)	0.51	(0.30, 0.87)	0.49	(0.26, 0.92)
3.20–3.49	0.70	(0.44, 1.10)	0.60	(0.37, 0.98)	0.98	(0.67, 1.43)	0.97	(0.66, 1.43)
3.50–3.79	0.90	(0.66, 1.24)	0.89	(0.63, 1.25)	0.96	(0.67, 1.37)	0.95	(0.64, 1.39)
3.80+	1	(ref)	1	(ref)	1	(ref)	1	(ref)
Phosphate binder use								
None	1	(ref)	1	(ref)	1	(ref)	1	(ref)
Ca-containing only	1.02	(0.59, 1.75)	1.21	(0.68, 2.15)	0.90	(0.55, 1.48)	0.91	(0.53, 1.54)
Non-Ca-containing only	1.30	(0.83, 2.04)	1.37	(0.82, 2.28)	1.55	(1.07, 2.24)	1.50	(1.06, 2.12)
Both	1.15	(0.72, 1.85)	1.22	(0.69, 2.15)	1.19	(0.75, 1.89)	1.20	(0.72, 1.99)
Vitamin D use								
None	1	(ref)	1	(ref)	1	(ref)	1	(ref)
IV vitamin D only	1.05	(0.72, 1.53)	0.95	(0.64, 1.43)	0.91	(0.61, 1.35)	0.87	(0.57, 1.33)
Oral vitamin D only	1.16	(0.81, 1.67)	1.06	(0.75, 1.50)	1.01	(0.71, 1.43)	0.94	(0.67, 1.31)
Both	0.86	(0.37, 2.02)	0.97	(0.46, 2.02)	0.61	(0.18, 2.08)	0.70	(0.21, 2.30)

^a^Adjusted for baseline covariates: age, vintage, 13 summary comorbid conditions, body mass index, and male sex. Separate models estimated for each variable shown. Time-dependent effects estimated only for variables shown. Psychiatric disorder includes depression, bipolar disorder, schizophrenia, and alcohol or substance abuse

### Post-discontinuation trends in CKD-MBD biomarkers

**Fig. 4:**
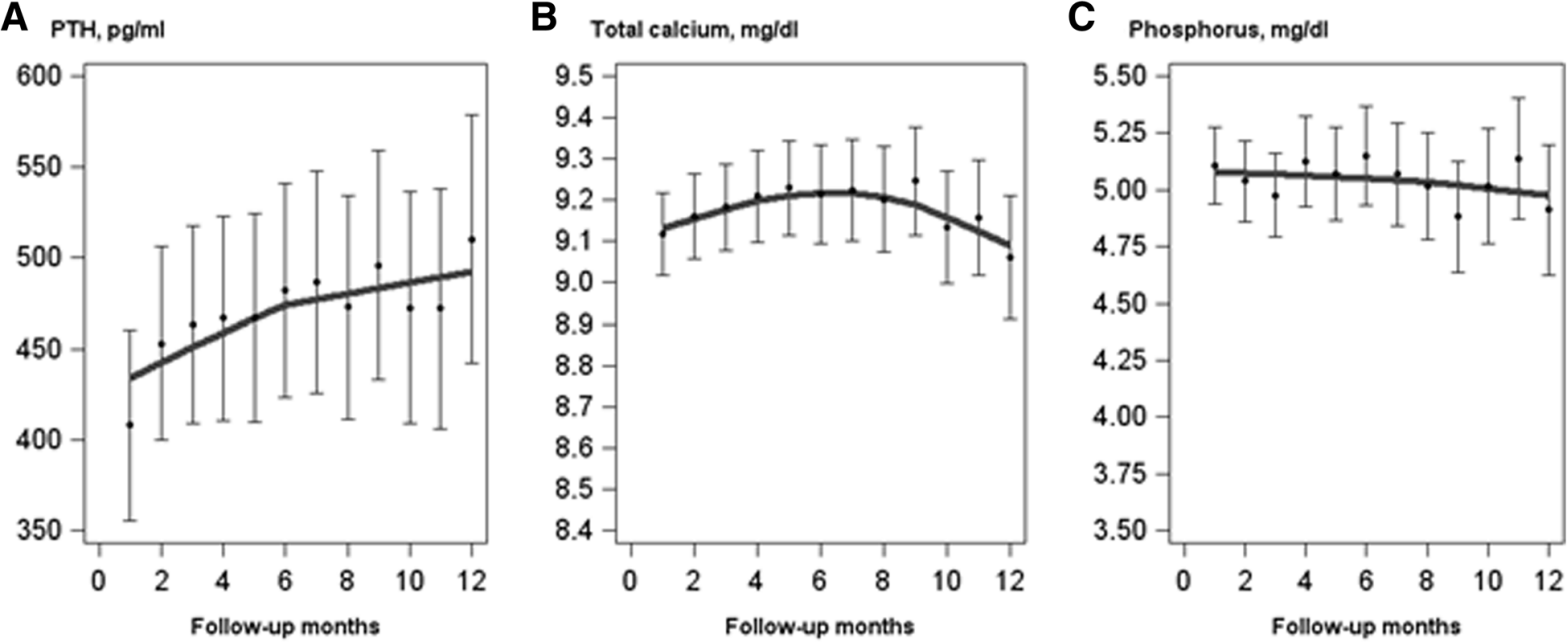
Observed monthly means and LOESS (LOcal regrESSion, or locally-weighted scatterplot smoothing) of **a** parathyroid hormone (PTH, *n* = 357), **b** total calcium (*n* = 346), and **c** phosphorus (*n* = 366) in the newly-discontinued-user cohort. Follow-up time began with the first monthly record after the discontinuation and was censored at reinitiation of cinacalcet treatment (prescription) or loss to follow-up for any reason. Errors bars denote 95% confidence intervals for the mean at each month

During the 12 month follow-up period after cinacalcet discontinuation, mean serum PTH levels increased, from 408 pg/ml (95% CI = 356–460) to 510 pg/ml (95% CI = 442–579) in the newly-discontinued-user cohort (Fig. 4; data table in Additional file 1: Table S1). Mean serum calcium levels increased from 9.12 mg/dl (95% CI = 9.02–9.22) to 9.22 mg/dl (95% CI = 9.10–9.35) within the first 6 months before declining to 9.06 mg/dl (95% CI = 8.91–9.21) at month 12. Throughout follow-up, mean serum phosphorus levels remained in the range of 4.9–5.1 mg/dl. Analyses restricted to patients with 12 months of cinacalcet-free follow-up did not reveal substantial differences in these trends.

## Discussion

We found that cinacalcet prescriptions were discontinued in 21 and 35% of European hemodialysis patients within 6 and 12 months, respectively. Among patients who discontinued cinacalcet after at least 3 months of prescription, nearly 50% returned to therapy within 12 months. We observed strong associations of levels of PTH and serum calcium in the previous month with both cinacalcet prescription discontinuation and reinitiation, but such associations were not observed with most variables measured at baseline. Patients with a discontinued cinacalcet prescription experienced a sustained subsequent increase in mean PTH levels, a small and temporary increase in serum calcium levels, and no change in serum phosphorus levels.

Our estimates of the proportion of patients with discontinued or reinitiated cinacalcet prescription are similar to other reports from Europe and the U.S. based on prescriptions entered into the medical record. In the US, Kilpatrick et al. [22] reported 20 and 30% cinacalcet discontinuation at 6 and 12 months, respectively, and 50% for reinitiation at 12 months. In a multi-center cohort of European hemodialysis patients treated at a single chain provider, de Francisco et al. [25] observed a slightly lower rate of discontinuation, 23% of patients within 1 year, based on a different definition of discontinuation (defined as > 45 prescription-free days). However, our results more closely align with their sensitivity analyses in which discontinuation was identified as the start of a 90-day period in which at least 60 days were prescription-free. Using the alternate definition, they found approximately 35% of patients discontinued cinacalcet within 1 year.

In the 12 months after discontinuation of cinacalcet, we observed a sustained trend of increasing PTH levels that stands in contrast to the modest increase (with gradual return to baseline) in calcium levels and generally stable phosphorus levels. Although we did not attempt to adjust our estimates of post-discontinuation CKD-MBD laboratory value trajectories for changes in IV or oral vitamin D agents that would influence laboratory values and might trigger cinacalcet reinitiation (and therefore censoring from the analysis), we found no substantial differences in laboratory value trajectories when excluding patients who restarted cinacalcet within 12 months. Our laboratory findings are consistent with the results of a European cohort study of incident hemodialysis patients from 2007 to 2009 that showed a sustained (but faster) PTH increase over 12 months and similarly modest trends in calcium and phosphorus levels [25]. In contrast, a study of US Medicare claims from 2007 to 2010 linked with clinical data from a large dialysis provider reported an initial PTH increase after cinacalcet discontinuation that returned to baseline, and an increase in calcium levels that was sharp and sustained [24]. The reasons for this juxtaposition of results are unclear. A patient’s failure to take cinacalcet as prescribed (non-adherence), which was not directly captured in our study, would result in delayed capture of removal of cinacalcet from the patient’s medication list (discontinuation, as defined in our study). In this case, the immediate effect of discontinuing cinacalcet on levels of CKD-MBD biomarkers may have already occurred by the observed time of discontinuation, thus  diminishing the potential for observing stronger post-discontinuation trends. Additionally, the observed trends may be impacted by our choice to censor patients at the reinitiation of cinacalcet; patients with greatly increased PTH levels would be excluded in later follow-up months, potentially restricting the observable magnitude of change among the remaining patients. We also note that our study includes more recent data through 2015 that may reflect changes in the international CKD-MBD clinical practice guidelines for SHPT management published in 2009 that liberalized the upper maintenance range for PTH [7, 26].

In our study, we found time-varying PTH levels to be highly associated with discontinuation and reinitiation of cinacalcet. Our finding of an increased rate of discontinuation at PTH < 150 pg/ml is consistent with the lower PTH target suggested by current international guidelines for hemodialysis patients (2 to 9 times the upper normal limit, approximately 130–585 pg/ml) [25] and with approved prescribing information for cinacalcet [10]. Similarly, our findings of an increasingly stronger association with increasingly higher PTH levels above 300 pg/ml with cinacalcet reinitiation align with PTH upper limits in European countries in the DOPPS. In DOPPS 5, 89% of facilities reported using an upper limit of 300 pg/ml or higher with a median upper limit among all facilities of 450 pg/ml [7]. Very low serum calcium levels < 7.5 mg/dl though uncommon were associated with a higher adjusted rate of discontinuation (HR: 1.83; 95% CI: 0.83–4.04) compared to patients having a serum calcium level of 8.4–10.2 mg/dl. Higher risk of discontinuation was also seen for moderately low calcium levels between 7.5 and 8.4 mg/dl (HR: 1.65, 95% CI: 1.04–2.60). Hypocalcemia is a contraindication for cinacalcet prescription [10], but a report from Brunelli, et al. suggested that in most patients, calcium levels typically returned to normal levels within 90 days regardless of directed therapeutic intervention [9]; see also Floege et al. [27].

Patient factors may also increase the likelihood that cinacalcet therapy is discontinued. Physicians may discontinue (or choose not to reinitiate) cinacalcet for symptomatic patients who appear to be malnourished or undernourished (e.g. based upon low serum phosphorus or albumin levels) due to gastrointestinal side effects associated with cinacalcet use. Unfortunately, our study did not collect longitudinal data on these side effects that could be used to assess their direct (i.e., non-adherence) and/or indirect (e.g. the patient reports negative side effects to the physician, who then terminates the prescription) influence on cinacalcet discontinuation. Additionally, we found that patients hospitalized within the prior 3 months both discontinued cinacalcet more often (HR = 1.40; 95% CI = 1.01–1.92) and reinitiated less often (HR = 0.82; 95% CI = 0.54–1.24). It is possible that during these periods of time, medication reconciliation is suboptimal and lapses occur for the refilling of oral medications, including cinacalcet. Thus, targeted use of home medication monitoring programs and cross-provider strategies to promote continuity of care during episodes of hospitalization may reduce the opportunity for PTH values to vary outside of target levels [28, 29]. Furthermore, the recent availability of etelcalcetide [30], a second-generation calcimimetic, can provide physicians with an option for reducing PTH levels as well or better than cinacalcet. Etelcalcetide is intravenously administered at the end of the dialysis session, potentially helping to improve adherence [31].

Our study benefits from its nationally representative selection of dialysis facilities and patients to provide generalizable and contemporary results for hemodialysis practice across seven European countries. However, our data collection protocol was limited to prescription information available in a patient’s medical chart. Thus, we were unable to directly identify patient-initiated discontinuation, including failure to take medication at prescribed dose or frequency (i.e., non-adherence) or failure to fill or refill a cinacalcet prescription, that occurred before the cinacalcet prescription was removed from the medication list. Likewise, our monthly ascertainment of cinacalcet prescription as a binary variable (active/absent) conservatively assumes an active prescription covered the entire month. Therefore, our results likely underestimate the true rates of cinacalcet discontinuation and the potential magnitude of post-discontinuation trends in CKD-MBD biochemistries.

## Conclusions

In summary, discontinuation of cinacalcet prescription, based on dialysis facility medical records, is common in European countries, occurring in approximately 35% of patients within 1 year. Given the observed rise in PTH after cinacalcet discontinuation, additional research is needed to identify optimal cinacalcet treatment strategies for SHPT management, including a comparison of intermittent cinacalcet therapy versus sustained treatment with reduced dose or frequency.

## Additional file

Additional file 1: **Table S1.** Data table (means and 95% confidence limits) for Fig. 4. (DOCX 14 kb)

## Abbreviations

BMI: Body mass index
CKD: Chronic kidney disease
DOPPS: Dialysis Outcomes and Practice Patterns Study
ESRD: End-stage renal disease
LOESS: LOcal regrESSion
MBD: Mineral and bone disorder
PTH: Parathyroid hormone
SHPT: Secondary hyperparathyroidism
UK: United Kingdom
