# Supplementary material for: Predictors of cinacalcet discontinuation and reinitiation in hemodialysis patients: results from 7 European countries
Source: BMC Nephrol. 2019 May 14;20:169. doi: 10.1186/s12882-019-1355-5 (PMC6518810; doi:10.1186/s12882-019-1355-5)
Supplement: Supplementary file 1 — Table S1. Data table (means and 95% confidence limits) for Fig. 4. (DOCX 14 kb) [file 12882_2019_1355_MOESM1_ESM.docx]

Table S1. Data table (means and 95% confidence limits) for Figure 4.

| **Months after discontinuation** | **PTH (pg/ml)** | **Total calcium (mg/dl)** | **Phosphorus (mg/dl)** |
| --- | --- | --- | --- |
| **1** | 408 (356-460) | 9.12 (9.02-9.22) | 5.11 (4.94-5.28) |
| **2** | 453 (400-506) | 9.16 (9.06-9.26) | 5.04 (4.86-5.22) |
| **3** | 463 (409-518) | 9.18 (9.08-9.29) | 4.98 (4.79-5.16) |
| **4** | 467 (411-523) | 9.21 (9.10-9.32) | 5.12 (4.92-5.32) |
| **5** | 467 (410-525) | 9.23 (9.11-9.34) | 5.07 (4.86-5.28) |
| **6** | 482 (423-541) | 9.21 (9.10-9.33) | 5.15 (4.93-5.37) |
| **7** | 487 (426-548) | 9.22 (9.10-9.35) | 5.07 (4.84-5.30) |
| **8** | 473 (411-535) | 9.20 (9.07-9.33) | 5.02 (4.78-5.25) |
| **9** | 496 (433-559) | 9.25 (9.11-9.38) | 4.88 (4.63-5.13) |
| **10** | 473 (409-536) | 9.13 (9.00-9.27) | 5.01 (4.76-5.27) |
| **11** | 472 (406-538) | 9.16 (9.02-9.30) | 5.14 (4.87-5.40) |
| **12** | 510 (442-579) | 9.06 (8.91-9.21) | 4.91 (4.62-5.20) |
